# Selective Secretion of KDEL-Bearing Proteins: Mechanisms and Functions

**DOI:** 10.3389/fcell.2022.967875

**Published:** 2022-07-13

**Authors:** F. C. Palazzo, R. Sitia, T. Tempio

**Affiliations:** Division of Genetics and Cell Biology Vita-Salute San Raffaele University and IRCCS San Raffaele Scientific Institute, Milan, Italy

**Keywords:** KDEL receptors, protein quality control, protein secretion, ERp44, PDI, protein folding, endoplasmic recticulum, Golgi

## Abstract

In multicellular organisms, cells must continuously exchange messages with the right meaning, intensity, and duration. Most of these messages are delivered through cognate interactions between membrane and secretory proteins. Their conformational maturation is assisted by a vast array of chaperones and enzymes, ensuring the fidelity of intercellular communication. These folding assistants reside in the early secretory compartment (ESC), a functional unit that encompasses endoplasmic reticulum (ER), intermediate compartment and cis-Golgi. Most soluble ESC residents have C-terminal KDEL-like motifs that prevent their transport beyond the Golgi. However, some accumulate in the ER, while others in downstream stations, implying different recycling rates. Moreover, it is now clear that cells can actively secrete certain ESC residents but not others. This essay discusses the physiology of their differential intracellular distribution, and the mechanisms that may ensure selectivity of release.

## The Assembly Line for Secretory Proteins

Secreted proteins are key messengers in cell-to-cell communication. The composition of the secretome depends on selective cargo export and retention/retrieval mechanisms. Key players in the latter are KDEL receptors (KRs), present in three isoforms in mammals. Through the recognition of C-terminal sequence KDEL, or variants thereof (e.g., KTEL, RDEL, HDEL), they prevent the secretion of the many and very abundant soluble chaperones and enzymes (Munro and Pelham, 1987), that assist the folding of a diverse secretome. These folding assistants operate in the early secretory compartment (ESC), a term we use herein to define the ER, intermediate compartment and cis-Golgi. Its stepwise organization is perfectly suited to time the many posttranslational modifications that cargoes need to attain their native structure.

**FIGURE 1:**
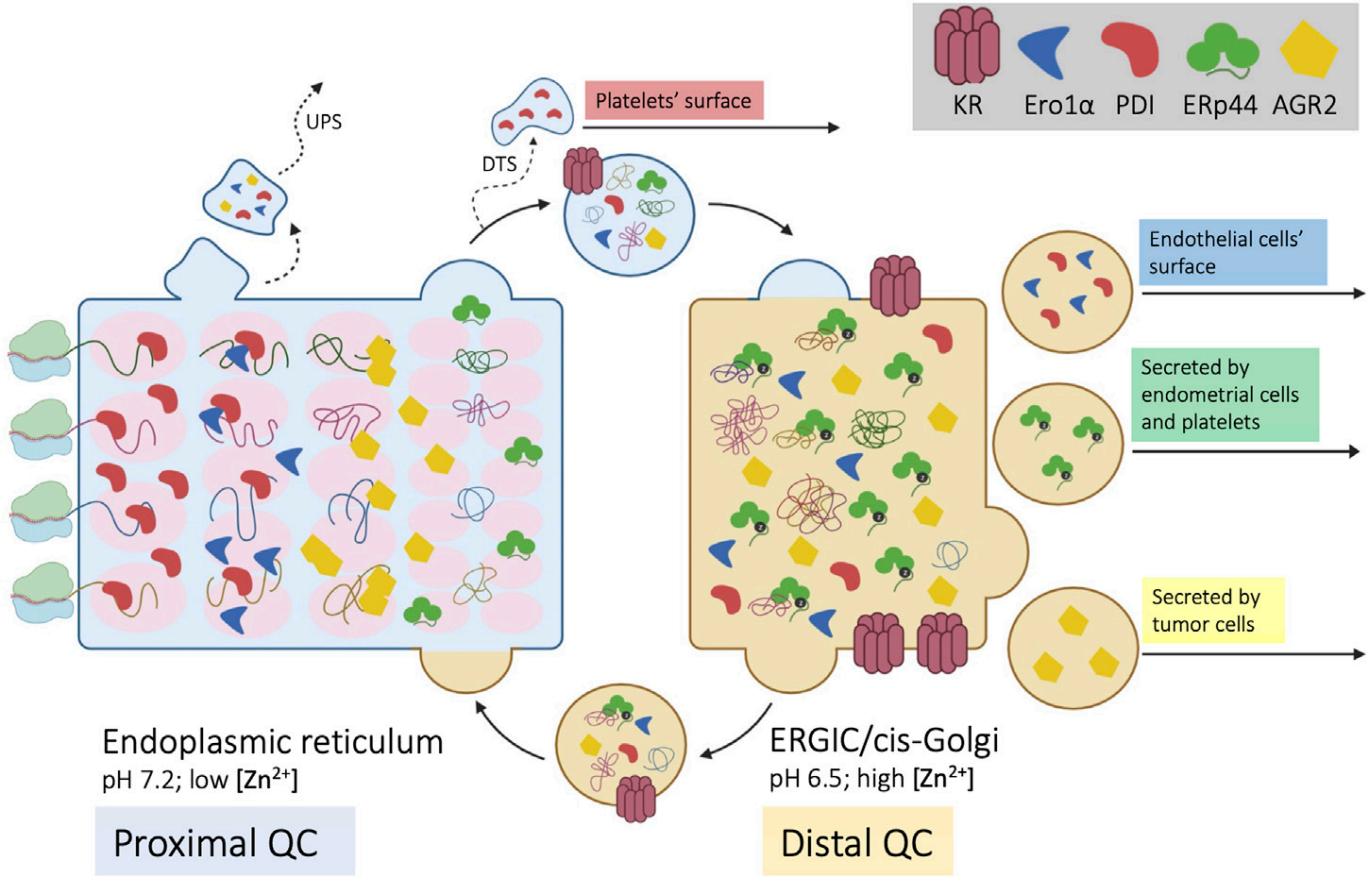
Self-sorting of chaperones and cargoes along the early secretory compartment (ESC). Four nascent cargo proteins are depicted on the left side of the cartoon as they cotranslationally translocate into the ER. Here, they encounter a series of chaperones and enzymes, that help their folding and prevent aggregation (proximal QC). The pink ovals represent supramolecular complexes with different diffusibility, with which cargo proteins sequentially interact. Once they reach a native, compact conformation, cargoes no longer interact with the matrix and can proceed towards downstream stations of the secretory pathway. ESC residents that reach the Golgi are instead retrieved by KRs. Unlike PDI, ERp44 reaches faster the distal compartment (ERGIC/cis-Golgi) to patrol the assembly of complex, multimeric proteins (distal QC). ERp44 retrieves also Ero1α and other enzymes that lack KDEL motifs (Prx4, ERAP1, FGE/SuMF1). Some proteins might be retro-translocated from the ER to the cytosol and exported via unconventional pathways of secretion (UPS). As shown in the right part of the cartoon, some ESC residents elude KRs’ retention/retrieval and are found also extracellularly. During megakaryocyte maturation, PDI is routed towards smooth ER fragments called “dense tubular system” (DTS). PDI and Ero1α are also found on the surface of endothelial cells, whilst ERp44 is secreted during decidualization of primary endometrial cells. AGR2 is instead abundantly secreted by tumor cells or upon inflammation.

Cargo proteins proceed along the ESC like cars in assembly lines: yet, workers must stay in place to execute their tasks in a precise order. How can few KRs retain the numerous resident proteins? And how are the latter distributed in ESC to operate in the optimal sequence along the folding-assembly line? Compelling evidence shows that KDEL-bearing proteins distribute differently along ESC, depending on the balance between retention and forward movement, in a self-assembling multiple phase separation system (Gilchrist et al., 2006; Anelli et al., 2007; Tempio et al., 2021). This structure allows sequential checkpoints in the biogenesis and quality control (QC) of complex cargo proteins. Figure 1 summarizes our hypothetical model of ESC as a chromatography column (Anelli et al., 2015) packed with supramolecular complexes of chaperones and enzymes (Booth & Koch, 1989; Meunier et al., 2002). The propensity of resident proteins to interact with each other, as well as redox oscillations, pH differences and increasing/decreasing concentration of salts and metals might determine the size of the beads, which would distribute accordingly, like the stones that remain in a sieve. In their route to secretion, newly synthesized, unfolded cargo proteins interact with the column matrices. On the one hand, these interactions favor folding and prevent aggregation of cargo proteins. On the other, they can further increase the matrix size, making the retention system hardly saturable (Reddy et al., 1996; Meunier et al., 2002).

Only compact cargoes that no longer interact with the column matrices can proceed further. For many complex cargoes, however, the folding schedule is not complete within the proximal QC checkpoints. Thus, some chaperones and enzymes evade the ER and patrol the arrival of complex molecules in the ERGIC and cis-Golgi compartments, operating a distal QC. For example, IgM and adiponectin are transported further downstream to oligomerize under the assistance of ERp44 (Hampe et al., 2015; Anelli and Sitia 2018; Giannone et al., 2022). Indeed, despite having a RDEL motif, ERp44 accumulates distally with respect to other ESC residents, like PDI. The faster rate at which it exits from the ER depends in part on interactions with the cargo receptor ERGIC53 (Tempio et al., 2021). Moreover, unlike PDI, ERp44 is not part of the BiP containing primary matrix (Meunier et al., 2002).

To further complicate the issue, some proteins acting primarily in the ER do not contain known localization signals. An outstanding example is Ero1α, a glycosylated flavoenzyme playing a crucial role in oxidative protein folding (Cabibbo et al., 2000; Mezghrani et al., 2001). Interestingly, while the yeast Ero1p contains an essential C-terminal tail mediating its association with the ER membrane (Pagani et al., 2001), this tail is absent from human Ero1α and Ero1β. As a matter of fact, to be retained/retrieved the two flavoproteins hijack the KDEL and RDEL motifs of PDI and ERp44 with whom they associate. The loss of an intrinsic localization motif from Ero1α and Ero1β likely reflects the need to perform new functions in metazoan with respect to unicellular yeast. In stressed mammalian cells, for instance, Ero1α can reach the Golgi, increase its oxidase activity upon phosphorylation by Fam20C, and return to the ER with ERp44 (Anelli et al., 2003; Zhang et al., 2018) or be secreted by certain cell types (Swiatkowska et al., 2010).

This scenario depicts a continuous flow of proteins in ESC that self-sort in functional order. Some, more “static”, are part of slowly diffusing supramolecular complexes with a low propensity to exit the ER (Figure 1). Others, more “dynamic”, cycle rapidly between ER and Golgi. Prototypes of the two are PDI and ERp44. Intriguingly, both retain Ero1α and are released by certain cell types, playing important functions extracellularly. Hereafter, we’ll use the term secretion to indicate active and specific transport, whilst release will encompass also non-specific mechanisms that involve loss of membrane integrity.

## What Determines the Exit of ESC Proteins?

### Loss of Membrane Integrity

Passive release upon severe stress, necrosis or pyroptosis could make ESC resident proteins a subset of damage-associated molecular pattern (DAMPs) molecules. These non-specific mechanisms can hardly allow the selective externalization of single or groups of molecules, even though being part of large multimolecular complexes might retard diffusion. This could yield a temporal hierarchy of release from damaged cells, in this way informing the organism of the nature of the incumbent danger.

### Specific Release of ESC Residents

**FIGURE 2:**
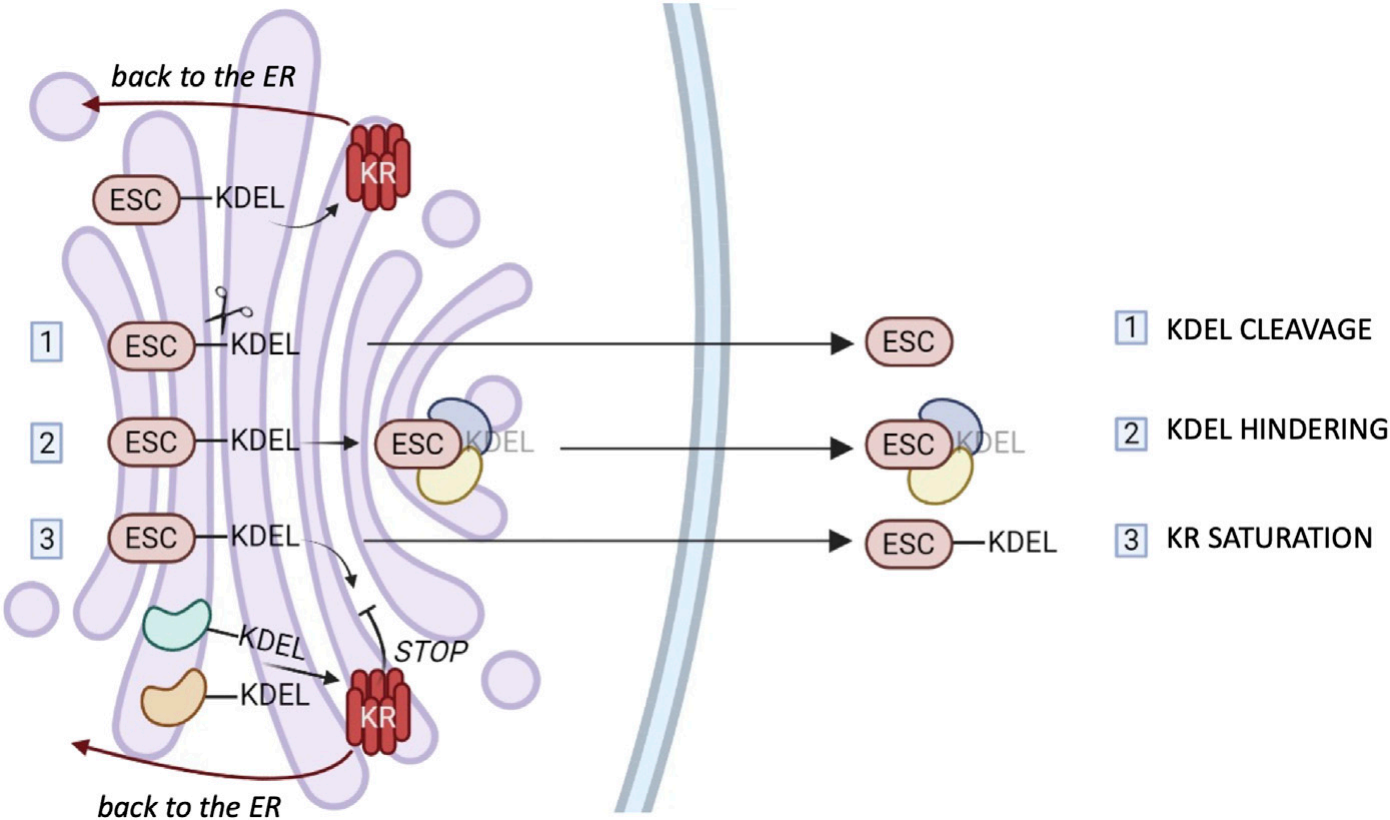
ESCape mechanisms. Most ER chaperones and enzymes are characterized by the presence of a C-terminal motif KDEL or variants thereof which mediates their retrieval to the ER via binding to one of the three KDEL receptors (KR). The cartoon summarizes possible mechanisms that these proteins might exploit to elude KRs’ surveillance. 1) Selective secretion by cleavage of the KDEL retention motifs. 2) Selective secretion upon binding of bulky clients that hinder KDEL-motifs. 3) Release upon weak KR retrieval activity due to client overload and/or Golgi basification. The different tissue distribution or preferential saturation/inhibition of individual KRs might sustain the selective secretion of chaperones or groups of them (see text for references).

How can ESC residents bearing KDEL motifs escape KR surveillance? Several mechanisms can be hypothesized, and they are summarized in Figure 2.

First of all, a selective proteolysis might remove the KDEL-like motif (step I in Figure 2). However, convincing evidence for this mechanism is missing.

Secondly, there could be the hindering of retrieval motifs by bulky clients (step II), proof of which has been obtained in the case of calreticulin (Johnson et al., 2001).

Moreover, considering that KRs are pH-sensitive (Bräuer et al., 2019), basification of the Golgi stacks might weaken their overall activity. Client overload, also, could transiently saturate KR-dependent retrieval systems (step III), but neither of these mechanisms would be sufficient to explain selective secretion of chaperones. In our hands, even when very abundantly overexpressed, ERp44 was not secreted, nor did it induce the secretion of other residents (Anelli et al., 2003; Vavassori et al., 2013). The structural similarities of the three KR argue against gross differences in their binding affinities for clients. Yet, the three human KRs are likely to serve different functions. Accordingly, their relative abundance changes in different tissues or cells (www.proteinatlas.org). However, it is still unclear whether they bind preferentially certain clients in the lumen, or different signaling molecules in the cytosol (Cancino et al., 2013; Luini et al., 2014). More detailed comparisons might reveal mechanisms that allow ER resident secretion by selective inhibition (or saturation) of a single KR. Intriguingly, in some cell types, KR1 can reach the plasma membrane, supposedly through clathrin-dependent transport carriers (Jia et al., 2021), in association with PDI, ERp57 and ERp5 (Bartels et al., 2019). It remains to be seen what allows KR1 to travel further and how its client oxidoreductases elude the other KRs. Specific secretion could be achieved if selected KDEL-bearing proteins arrive with forward-moving cargo transporters, as described for ERp44 (Tempio et al., 2021).

### Unconventional Secretory Pathway

Some proteins that lack a leader sequence (e.g., IL-1β, FGF2 or thioredoxin) are actively secreted by living cells via diverse pathways (Sitia & Rubartelli, 2020). In many cases, their precursors can translocate into lysosomes, or reach them by autophagic pathways (Duran et al., 2010; Dupont et al., 2011). Particularly in hematopoietic cells, lysosomes can fuse with the plasma membrane, releasing their contents in the extracellular space. It is possible that also ESC residents follow this route. Concentration of soluble proteins in ESC subregions might recruit autophagosomes leading to selective secretion.

Moreover, in conditions of ER stress, prion protein and calreticulin are shunted to the cytosol in the process of pre-emptive quality control (Orsi et al., 2006; Snapp et al., 2006). It has also been proposed that, upon harsh ER stress, some ER chaperones can be translocated to the cytosol in a process defined “ER reflux” (Igbaria et al., 2019; Sicari et al., 2021). Then, from the cytosol, these proteins might also follow chaperone-mediated unconventional secretory pathways to reach the extracellular space.

## Extracellular Functions of ESC Residents

The “selective secretion” of proteins that are normally retained inside may represent a novel code that cells use to interact with each other. When secreted, ESC residents can interfere directly or indirectly with ligands, receptors and the signals generated by their interactions. In any case, to reflect specific pathophysiologic conditions their release should be regulated in time and space.

Although several lines of evidence suggest new roles for “mis-localized” ESC residents, the transport mechanisms and functions are still controversial. Even in systems in which selective secretion is documented, only a minor fraction of the ESC resident in question is found extracellularly (Soares Moretti and Martins Laurindo, 2017). This does not diminish the potential pathophysiological implications, though. Considering the almost virtual space in immunological and neural synapses and between cells in contact, even few molecules may reach concentrations sufficient to exert relevant biological phenomena. Rarely does evolution leave important pathways to chance: hence, the release of ESC components by living and unstressed cells would be most likely linked to tightly regulated paths.

### Blood Clotting

There is ample evidence that the presence of PDI in the extracellular space impacts blood clotting, virus entry and other pathophysiological events (Gallina et al., 2002; Markovic et al., 2004; Furie & Flaumenhaft, 2014; Fraternale et al., 2021; Xu et al., 2021). In the ER, PDI is present in high concentration, up to 0.2–0.5 mM (Lyles & Gilbert, 1991; Laurindo et al., 2012), making it a well-known marker of this compartment.

Its presence in the medium and on the surface of some cell types has been widely reported (Araujo et al., 2017; Wu & Essex, 2020). During vascular injury, for example, activated platelets and endothelial cells release PDI in the extracellular space (Cho et al., 2012; Bowley et al., 2017; Sharda & Furie, 2018). Moreover, they present also PDI molecules on their surface, which interact with β_3_ integrins (Cho et al., 2012; Ponamarczuk et al., 2018). Given its role in blood clotting, one would expect PDI secretion being mostly short range, essentially of paracrine nature.

Upon platelet activation, also other thiol isomerases, such as ERp5, ERp57, ERp72, ERp44 and ERp29 are exported into the medium or bound to the outer leaflet of the plasma membrane (Holbrook et al., 2010; Tanaka et al., 2020; Wu & Essex, 2020). Whether their export depends on aspecific release or active secretion is still debated. For instance, during megakaryocyte maturation, PDI and ERp57 molecules that will be found in platelets do not follow the normal Golgi-secretory vesicles route. Rather, they accumulate in a subcellular compartment called “dense tubular system”, which derives from the smooth ER of megakaryocytes (Crescente et al., 2016). Additionally, it has been proposed that stimulated endothelial cells secrete PDI in Gro-α-containing granules (Jasuja et al., 2010). Within these granules, PDI might hide its KDEL and elude retrieval, as described for calreticulin (Johnson et al., 2001).

Anyway, whatever is the transport mechanism, paramount are the pathophysiologic consequences of thiol isomerases appearance outside the cell.

### Potential Synergies in the Redox Control of Extracellular Thiol Isomerases

Not only their abundance but also the redox state of PDI and other oxidoreductases are bound to determine their extracellular function. PDI molecules found on activated platelets are mainly in their reduced state (Essex & Wu, 2018), while the reverse is true in vascular smooth muscle cells (Tanaka et al., 2016, 2019). In the ER, PDI oxidation depends largely on the selective binding of Ero1α to it’s a domain. Yet, part of PDI must remain reduced to mediate disulfide isomerization. In the extracellular space, its redox state depends on the presence of non-protein couples (e.g., GSH-GSSG) or redox enzymes such as thioredoxin, QSOX or Ero1α. Thus, other cell types can modulate the ultimate function of PDI and other oxidoreductases. The reversible modifications of the catalytic cysteines of these enzymes are powerful molecular switches. Thus, the presence of oxidants in constrained extracellular spaces is particularly relevant. For this reason, the detection of also Ero1α on the surface of activated platelets is not trivial (Swiatkowska et al., 2010). In the ER, and also in test tubes, Ero1α re-oxidizes PDI at the expense of O_2_ molecules (Appenzeller-Herzog et al., 2010). Therefore, their vicinity on the platelets surface would fuel oxidative equivalents that sustain clotting through disulfide bond formation or isomerization in extracellular substrates (Swiatkowska et al., 2010). As the ER localization of Ero1α depends also on ERp44, its release would involve at least two control levels. In cells actively secreting PDI, also the surveillance of ERp44 must be eluded by Ero1α. What if Ero1α is released by other cells in which ERp44 is inactive or itself secreted?

The localization and cycling of ERp44 are controlled by zinc- and pH-dependent conformational changes that expose its client binding site and RDEL motif (Vavassori et al., 2013; Watanabe et al., 2019; Tempio & Anelli, 2020). O-glycosylated ERp44 is secreted by primary and immortalized endometrial cells (Sannino et al., 2014) suggesting transport through the canonical secretory pathway. If not in all, in most cell types, lowering zinc concentration induces rather selectively the secretion of ERp44 and its clients. Upon pH manipulation, instead, ERp44 is retained while its cargos Ero1α, Prx4 and ERAP1 are secreted.

### Cancer and Inflammation

Like ERp44, the small thioredoxin family member AGR2 lacks the resolving cysteine in its active motif and is hence better suited for an isomerase/holdase than an oxidoreductase function (Moidu et al., 2020). Despite its KTEL motif warrants ESC residency, it has a reduced binding affinity for the KRs (Raykhel et al., 2007; Alanen et al., 2011). This could explain its well documented secretion in response to physiological and/or pathological conditions. Indeed, besides its established intracellular roles in the folding, trafficking, and assembly of mucins, EGF receptors and other cysteine-rich proteins (Delom et al., 2020; Moidu et al., 2020), AGR2 has been found secreted or bound to the plasma membrane (Chevet et al., 2013; Dumartin et al., 2017). In cancer, these extracellular localizations of AGR2 have been proposed to modulate matrix remodeling, metastasis progression and support angiogenesis (Delom et al., 2018). Enhanced AGR2 secretion by ER stressed cells is thought also to modulate pro-inflammatory signals, particularly in inflammatory bowel disease (Maurel et al., 2019). The presence of O-glycans in extracellular AGR2 suggests that it follows the canonical secretory route (Clarke et al., 2015; Tian et al., 2018; Moidu et al., 2020).

### Conclusions and Open Questions

After the finding that cells exchange bits of cytosol and membranes with each other, another pillar of cell biology fell apart with the detection on the plasma membrane or in the extracellular fluids of proteins traditionally used as markers of the ESC. Is this “extracellular folding compartment” a new way that cells use to communicate with each other? Are moonlighting and multitasking obligatory features of proteins in multicellular organisms? In this respect, we can imagine an intercellular compartment in which individual folding components are provided by different cells.

The presence of small redox compounds and enzymes in the extracellular space becomes crucial. For instance, stressed cells release cysteine, thioredoxin and other stress associated molecular pattern (SAMPs) molecules, often through unconventional secretory pathways, that dictate the intensity and duration of inflammatory responses (Sitia & Rubartelli, 2020; Yang et al., 2021). It is tempting to speculate that fundamental decisions, like thrombus initiation, depend on the simultaneous release of synergizing compounds by different cells.

A key issue is the mechanism(s) of secretion. Posttranslational modifications, glycan processing in particular, can provide powerful cues to decipher the route ESC residents follow to reach the extracellular space. Indeed, the release of O-glycosylated ERp44 and AGR2 argues in favor of passage through the Golgi. Since sugar bound asparagines are converted to aspartates by cytosolic glycanases, an accurate characterization of suitably engineered reporters might shed some light on this important issue. It is possible that multiple pathways exist that regulate the export of luminal ESC residents as biological signals, like in the case of the diverse unconventional pathways used by leaderless proteins (Sitia & Rubartelli, 2020).

An increased knowledge on these secretory pathways and their regulation may allow manipulating key pathophysiological circuits, including inflammation, angiogenesis, and blood clotting, in this way reestablishing homeostasis.

Overall, this could be also important to find new therapeutic targets for diseases still having great unmet medical needs.
